# Supplementary material for: Increased Preeclampsia Risk in GDM Pregnancies: The Role of SIRT1 rs12778366 Polymorphism and Telomere Length
Source: Int J Mol Sci. 2025 Mar 25;26(7):2967. doi: 10.3390/ijms26072967 (PMC11988573; doi:10.3390/ijms26072967)
Supplement: Supplementary file 1 [file ijms-26-02967-s001.zip › ijms-3498502-supplementary.pdf]

Supplementary Table S1: Mean telomere length depending on the genotypes rs12778366 and rs7895833 of the SIRT1 gene in pregnant women with GDM in the PE and Control groups.

Genotypes	Mean telomere length		
	**PE**	**Control**	***p*-value***
	***SIRT1* rs12778366**		
**TT**	0.89±0.07	0.92±0.04	0.104
**CT**	0.90±0.04	0.95±0.02	
	***SIRT1* rs7895833**		
**AA**	0.89±0.07	0.92±0.06	0.088
**AG**	0.90±0.04	0.91±0.03	
**GG**	0.87	0.94±0.02	

Descriptions for Supplementary Table S1: *Test test χ2
